# Comparison of emergency cholecystectomy and delayed cholecystectomy after percutaneous transhepatic gallbladder drainage in patients with acute cholecystitis: a systematic review and meta-analysis

**DOI:** 10.1007/s13304-020-00894-4

**Published:** 2020-10-13

**Authors:** Shao-Zhuo Huang, Hao-Qi Chen, Wei-Xin Liao, Wen-Ying Zhou, Jie-Huan Chen, Wen-Chao Li, Hui Zhou, Bo Liu, Kun-Peng Hu

**Affiliations:** 1 Department of General Surgery, The Third Affiliated Hospital, Sun Yat-sen University, Guangzhou, China; 2 Department of Hepatic Surgery, The Third Affiliated Hospital, Sun Yat-sen University, Liver Transplantation, Guangzhou, China; 3 Department of Infectious Diseases, The Third Affiliated Hospital, Sun Yat-sen University, Guangzhou, China; 4 Department of Laboratory Medicine, The Third Affiliated Hospital of Sun Yat-sen University, Guangzhou, China; 5 Department of Ultrasound, Binhaiwan Central Hospital of Dongguan, Dongguan, China

**Keywords:** Cholecystectomy, PTGBD, Acute cholecystitis, Meta-analysis

## Abstract

Laparoscopic cholecystectomy and percutaneous transhepatic gallbladder drainage (PTGBD) are common treatments for patients with acute cholecystitis. However, the safety and efficacy of emergency laparoscopic cholecystectomy (ELC) and delayed laparoscopic cholecystectomy (DLC) after PTGBD in patients with acute cholecystitis remain unclear. The PubMed, EMBASE, and Cochrane Library databases were searched through October 2019. The quality of the included nonrandomized studies was assessed using the Methodological Index for Nonrandomized Studies (MINORS). The meta-analysis was performed using STATA version 14.2. A random-effects model was used to calculate the outcomes. A total of fifteen studies involving 1780 patients with acute cholecystitis were included in the meta-analysis. DLC after PTGBD was associated with a shorter operative time (SMD − 0.51; 95% CI − 0.89 to − 0.13; *P* = 0.008), a lower conversion rate (RR 0.43; 95% CI 0.26 to 0.69; *P* = 0.001), less intraoperative blood loss (SMD − 0.59; 95% CI − 0.96 to − 0.22; *P* = 0.002) and longer time of total hospital stay compared to ELC (SMD 0.91; 95% CI 0.57–1.24; *P* < 0.001). There was no difference in the postoperative complications (RR 0.68; 95% CI 0.48–0.97; *P* = 0.035), biliary leakage (RR 0.65; 95% CI 0.34–1.22; *P* = 0.175) or mortality (RR 1.04; 95% CI 0.39–2.80; *P* = 0.933). Compared to ELC, DLC after PTGBD had the advantages of a shorter operative time, a lower conversion rate and less intraoperative blood loss.

## Introduction

Acute cholecystitis (AC), an inflammatory condition of the gallbladder, is also a common disease that involves hospitalization and surgical treatment [1]. Patients with AC may present with a wide spectrum of inflammation, which may progress to empyema, perforation, and abscess formation, with an overall mortality rate of approximately 0.6% [2–4]. Since the first cholecystectomy was performed with the use of an operative laparoscope 30 years ago [5], laparoscopic cholecystectomy (LC) is currently recognized as a standard treatment for AC. In most AC patients, laparoscopic cholecystectomy can rapidly attenuate inflammatory symptoms and signs. Nevertheless, LC may precipitate certain complications, such as biliary leakage, bile duct injury and intra-abdominal abscess [6, 7], especially in elderly AC patients who undergo emergency laparoscopic cholecystectomy, which may cause high morbidity and mortality rates [8]. Several studies have also documented that LC is associated with a high rate of conversion to open cholecystectomy and a long length of hospital stay [9, 10].

Percutaneous transhepatic gallbladder drainage (PTGBD), which was first applied by Radder in 1980 [11], is a minimally invasive operation performed to relieve gallbladder tension through external drainage under ultrasound or CT guidance [3]. According to the 2018 Tokyo guidelines for drainage management of AC, PTGBD can alleviate inflammation caused by edema of the gallbladder wall and pericholecystic adhesions, and it has the advantage of a lower risk of adverse events compared with cholecystectomy, which is an alternative to surgical treatment in high-risk AC patients [6, 12, 13]. Previous studies have reported that LC performed after PTGBD has several advantages, such as early symptom remission, surgery facilitation and patient stabilization [14–16]. However, PTGBD may also cause complications related to the procedure, such as bile leakage and pain at the puncture site [16, 17]. For severe AC patients, studies have shown that PTGBD is related to a high mortality rate and prolonged hospital stay [18]. In addition, biliary obstruction and cholestasis caused by gallstones are the major causes of acute cholecystitis, which is difficult to treat with PTGBD [19]. Besides, PTGBD may lead to complications associated with the drainage tube, such as obstruction of the drainage by stones and debris, pierced gallbladder and slipping off drainage tube [17].

The effects of emergency laparoscopic cholecystectomy (ELC) and delayed laparoscopic cholecystectomy (DLC) after PTGBD in AC patients remain unclear. Therefore, we conducted this meta-analysis to better understand and compare the safety and efficacy of ELC and PTGBD followed by DLC in AC patients in terms of the operative time, rate of conversion to open surgery, length of hospital stay, intraoperative blood loss, postoperative complications, biliary leakage and mortality.

## Materials and methods

### Data source

This study was carried out according to the Preferred Reporting Items for Systematic Reviews and Meta-Analyses 2009 guidelines [20]. Conducted by two authors (H.S. and C.H.), the search was performed using PubMed (1980 to October 2019), EMBASE (1988 to October 2019), and the Cochrane Central Register of Controlled Trials. The following medical subject terms were used in the search: (“cholecystectomy” OR “cholecystectomies”) AND (“drainage”) AND (“cholecystitis” OR “gallbladder Inflammation” OR “inflammation, gallbladder” OR “empyema, gallbladder” OR “gallbladder empyema” OR “empyema, gall bladder” OR “gall bladder empyema”). Neither IRB approval nor written consent was required for this study.

### Study selection

**Fig. 1:**
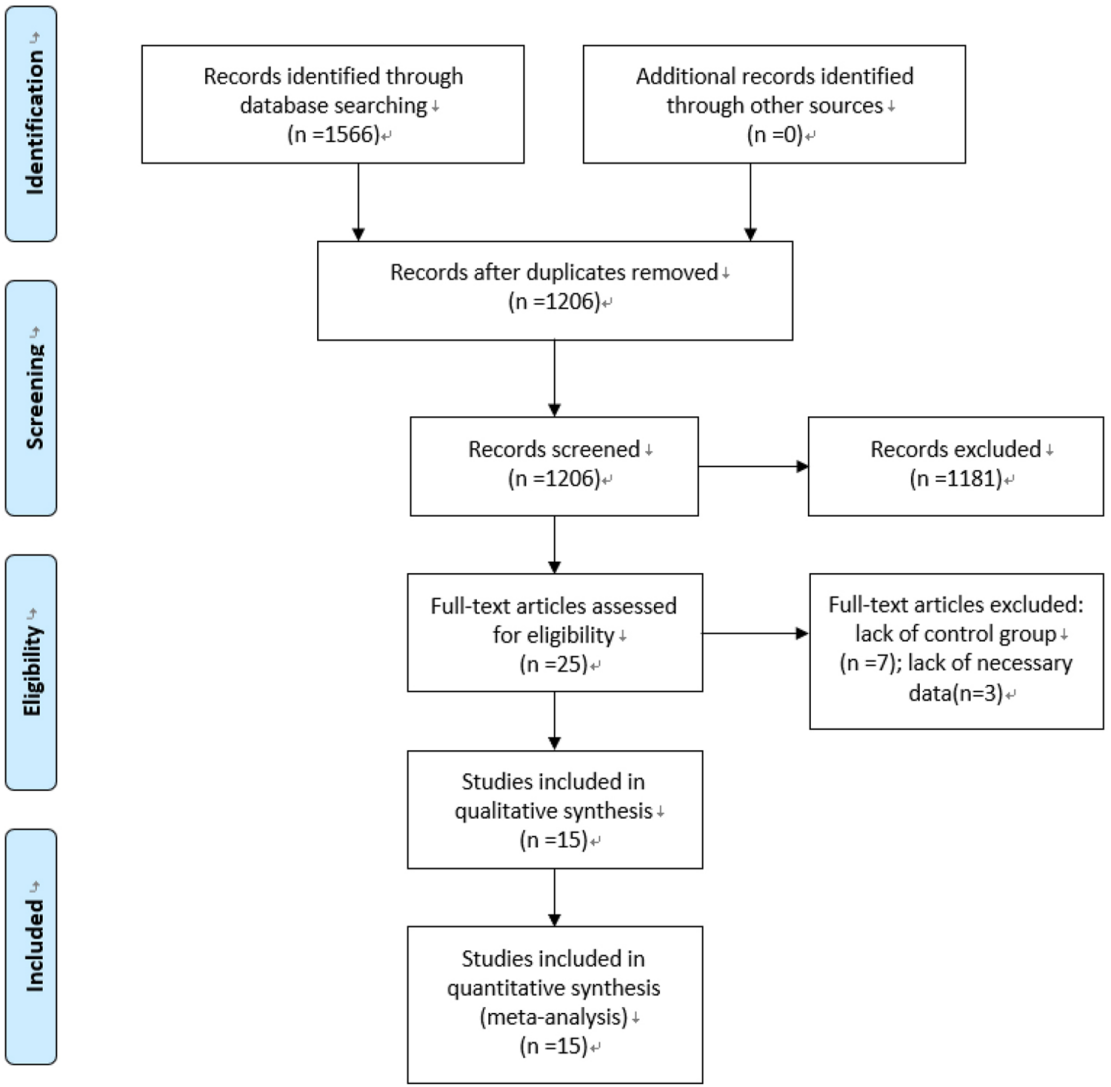
PRISMA flow diagram of literature screening

Duplicates were removed. Titles and abstracts were independently screened by the reviewers (H.S. and C.H.) to assess the relevance of the publications. Subsequently, full-text articles were retrieved and checked. The remaining articles were surveyed by cross-referenced searches to detect studies that might have been overlooked. All studies concerned patients treated with emergency cholecystectomy and delayed cholecystectomy after percutaneous transhepatic gallbladder drainage for acute cholecystitis. The criteria for the diagnosis of acute cholecystitis had to be defined in the article, and acute cholecystitis had to be proven either by ultrasound or histologically. No language or publication types were restricted. Studies only aimed at severe cases or special population were excluded. Studies lacking necessary data or control groups were excluded. In studies with multiple publications from the same population, only the most recent one was included. Letters, posters, conference abstracts, expert opinions, review articles, case reports, animal experiments, and in vitro studies were also excluded. The selection process of the studies is shown in Fig. 1.

### Data extraction

Data were extracted independently from each study by two investigators (H.S. and C.H.). The following items recorded for each study were extracted: first author, year of publication, age, country, number of controls and cases, study period, and time between PTGBD and LC. However, some studies expressed data with medians and ranges. We changed these data means with standard deviations [21]. Quantitative statistics were reported as the mean ± SD.

### Quality assessment

All of the studies were retrospective. The data quality of nonrandomized studies was assessed using the Methodological Index for Nonrandomized Studies (MINORS) (Table 1) [22]. MINORS, an index designed to assess the methodological quality of nonrandomized surgical studies, was developed by a group of surgeons because of the problems faced by clinicians as to the lack of randomized surgical trials and the large number of observational studies in surgery. By considering 12 items (8 for noncomparative and 4 for comparative studies), the total score was calculated by summing the values attributed as follows: 0 (not reported), 1 (reported but inadequate), and 2 (reported and adequate). The global ideal score for noncomparative studies was 16 and for comparative studies was 24.

**Table 1:** Quality assessment of non-randomized trials

Author (year)	Quality evaluation criteria								Additional criteria in comparative studies				
	Clear started aim	Inclusion of consecutive patients	Prospective data collection	Endpoints appropriate to the study aim	Unbiased assessment of study end point	Appropriate follow-up period	Loss to follow-up less than 5%	Prospective calculation of the study size	Adequate control group	Contemporary groups	Baseline equivalence	Adequate statistical analysis	Total
Kim (2000)	2	2	0	2	2	2	2	0	2	2	2	2	20
Chikamori (2002)	2	2	0	2	2	2	1	0	1	2	2	2	18
Tsumura (2004)	2	2	0	2	2	2	2	0	2	2	2	2	20
Kim (2008)	2	2	0	2	2	2	2	0	2	2	2	2	20
Kim (2009)	2	2	0	2	2	2	2	0	2	2	2	2	20
Kim (2011)	2	2	0	1	2	2	2	0	2	2	2	2	19
Choi (2012)	2	2	0	2	2	2	1	0	2	2	2	2	19
Hu (2015)	2	2	0	2	2	2	2	0	2	2	2	2	20
Na (2015)	2	2	0	2	2	2	2	0	2	2	2	2	20
Ni (2015)	2	2	0	2	2	2	2	0	2	2	2	2	20
EI-Gendi (2017)	2	2	0	2	2	2	2	0	2	2	2	2	20
Jung (2017)	2	2	0	1	2	2	1	0	2	2	2	2	18
Lee (2017)	2	2	0	1	2	2	1	0	2	2	2	2	18
Jia (2018)	2	2	0	2	2	2	2	0	2	2	2	2	20
Ke (2018)	2	2	0	2	2	2	2	0	2	2	2	2	20

Items are scored as follows: 0 (not reported), 1 (reported but inadequate), 2 (reported and adequate). Global ideal score for non-comparative studies is 16 and for comparative ones is 24

### Statistical analysis

Data from the eligible studies were extracted independently. Statistics Analysis (STATA version 14.2) (Stata Corporation; College Station, TX, USA) was used to perform the data analysis. Differences between groups were expressed as RRs with 95% CIs. The random-effect models were used to calculate the outcomes [23]. All statistical analyses used in this study were two-sided, and *P* < 0.05 was regarded as statistically significant.

Cochran’s *Q* test and *I*^2^ statistic were used to evaluate the heterogeneity across the studies. Cochran’s *Q* < 0.10 or *I*^2^ > 50% was regarded as significant heterogeneity across studies [24, 25]. To evaluate the extent of publication bias, Egger’s test and Begg’s test were used [26, 27]. Subgroup analysis was used to decrease the heterogeneity among studies.

## Results

### Literature retrieval and study selection

A total of 1566 articles were retrieved from the electronic databases (PubMed [*n* = 732], EMBASE [*n* = 817], and Cochrane [*n* = 17]). Cross-referenced searches did not find new articles. After removing duplicates (*n* = 360), the titles and abstracts of the remaining articles were examined. A total of 1181 articles were excluded because of irrelevancy. Subsequently, the remaining 25 articles were retrieved for detailed assessments based on the full texts. Among these 25 articles, 10 were excluded because of a lack of a control group (*n* = 7) and necessary data (*n* = 3). Finally, 15 articles were included in this meta-analysis [3, 4, 6, 10, 15–17, 28–35].

### Study characteristics and quality

The characteristics of the 15 included studies are shown in Table 2. Of the 15 included studies, 8 were conducted in Korea, 4 in China, and 2 in Japan, and the remaining study was conducted in Egypt. In addition, patients in six studies underwent LC after PTGBD within 7 days. Patients in eight studies underwent LC after PTGBD after 7 days. Specifically, one of the studies included both of these groups, so we split it into two groups: Kim① and Kim②.

**Table 2:** Characteristics of the included studies

Author	Year	Mean ± SD Age (years)	Country	Control/cases	Study period	Time between PTGBD and LC (days)
Kim	2000	ELC: 51 ± 13 PTGBD + DLC: 53 ± 12.5	Korea	45/27	1994–1999	< 7
Chikamori	2002	ELC: 67 ± 13 PTGBD + DLC: 65 ± 10	Japan	9/31	1998–2002	< 7
Tsumura	2004	ELC: 55.4 ± 16.7 PTGBD + DLC: 64.5 ± 13.6	Japan	73/60	1998–2003	> 7
Kim	2008	ELC: 60.5 ± 13.4 PTGBD + DLC: 66.8 ± 11.7	Korea	62/37	2003–2006	> 7
Kim①^*^	2009	ELC: 55.5 ± 13.3 PTGBD + DLC: 57.7 ± 11.9	Korea	60/35	2002–2007	< 7
Kim②^*^	2009	ELC: 55.5 ± 13.3 PTGBD + DLC: 61.0 ± 12.1	Korea	60/38	2002–2007	> 7
Kim	2011	ELC: 55.5 ± 13.3 PTGBD + DLC: 66.4 ± 15.3	Korea	147/97	2006–2009	< 7
Choi	2012	ELC: 60.4 ± 13.0 PTGBD + DLC: 72.5 ± 12.6	Korea	63/60	2007–2011	< 7
Hu	2015	ELC: 71.5 ± 11.5 PTGBD + DLC: 72.5 ± 12.6	China	35/35	2010–2014	> 7
Na	2015	ELC: 72.55 ± 7.00 PTGBD + DLC: 72.95 ± 7.49	Korea	77/39	2009–2013	< 7
Ni	2015	ELC: 59.0 ± 12.9 PTGBD + DLC: 65.6 ± 13.6	China	33/26	2005–2012	> 7
EI-Gendi	2017	ELC: 50.19 ± 12.01 PTGBD + DLC: 49.65 ± 11.63	Egypt	75/75	2014–2016	> 7
Jung	2017	ELC: 56.3 ± 15.5 PTGBD + DLC: 64.9 ± 14.9	Korea	166/128	2010–2014	> 7
Lee	2017	ELC: 61.6 ± 15.6 PTGBD + DLC: 69.0 ± 11.5	Korea	41/44	2013–2016	> 7
Jia	2018	ELC: 65.28 ± 16.71 PTGBD + DLC: 62.11 ± 13.1	China	48/38	2013–2015	< 7
Ke	2018	ELC: 62 ± 16 PTGBD + DLC: 67 ± 14	China	47/49	2013–2017	> 7

*SD* standard deviation, *PTGBD* percutaneous transhepatic gallbladder drainage, *LC* laparoscopic cholecystectomy, *ELC* emergency laparoscopic cholecystectomy, *DLC* delayed laparoscopic cholecystectomy

*Kim① and Kim② came from the same study that split into two groups according to the time between PTGBD and LC

Based on the quality assessment of MINORS, ten studies [3, 4, 10, 17, 28–33] scored 20 points, two studies [15, 34] scored 19 points, and three studies [6, 16, 35] scored 18 points. All studies had a nonrandomized design.

### Operative time of LC

**Fig. 2:**
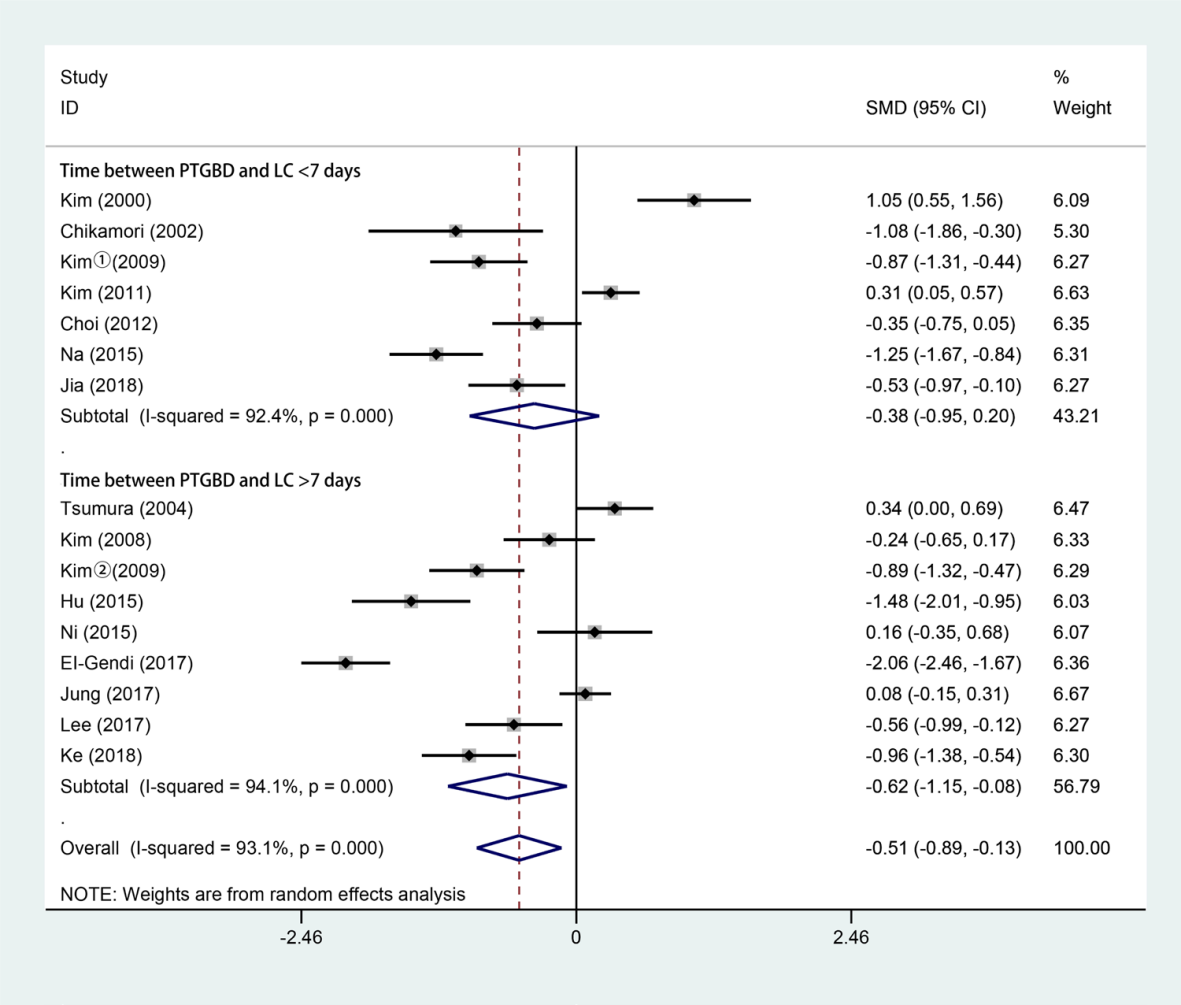
Forest plot of operative time of LC after PTGBD in patient with acute cholecystitis

All 15 studies [3, 4, 6, 10, 15–17, 28–35] reported the operative time of LC. All of these studies were analyzed by the mean with standard deviation. As shown in Fig. 2, 799 patients underwent LC after PTGBD, and 981 underwent ELC. According to the random-effects model, the pooled operative time of LC in the PTGBD group was shorter than that in the ELC group (SMD − 0.51; 95% CI − 0.89 to − 0.13; *P* = 0.008). There was a significant difference in heterogeneity among the studies (*Q* = 218.94; *P* for heterogeneity < 0.001; *I*^2^ = 93.1%).

### Conversion rate

**Fig. 3:**
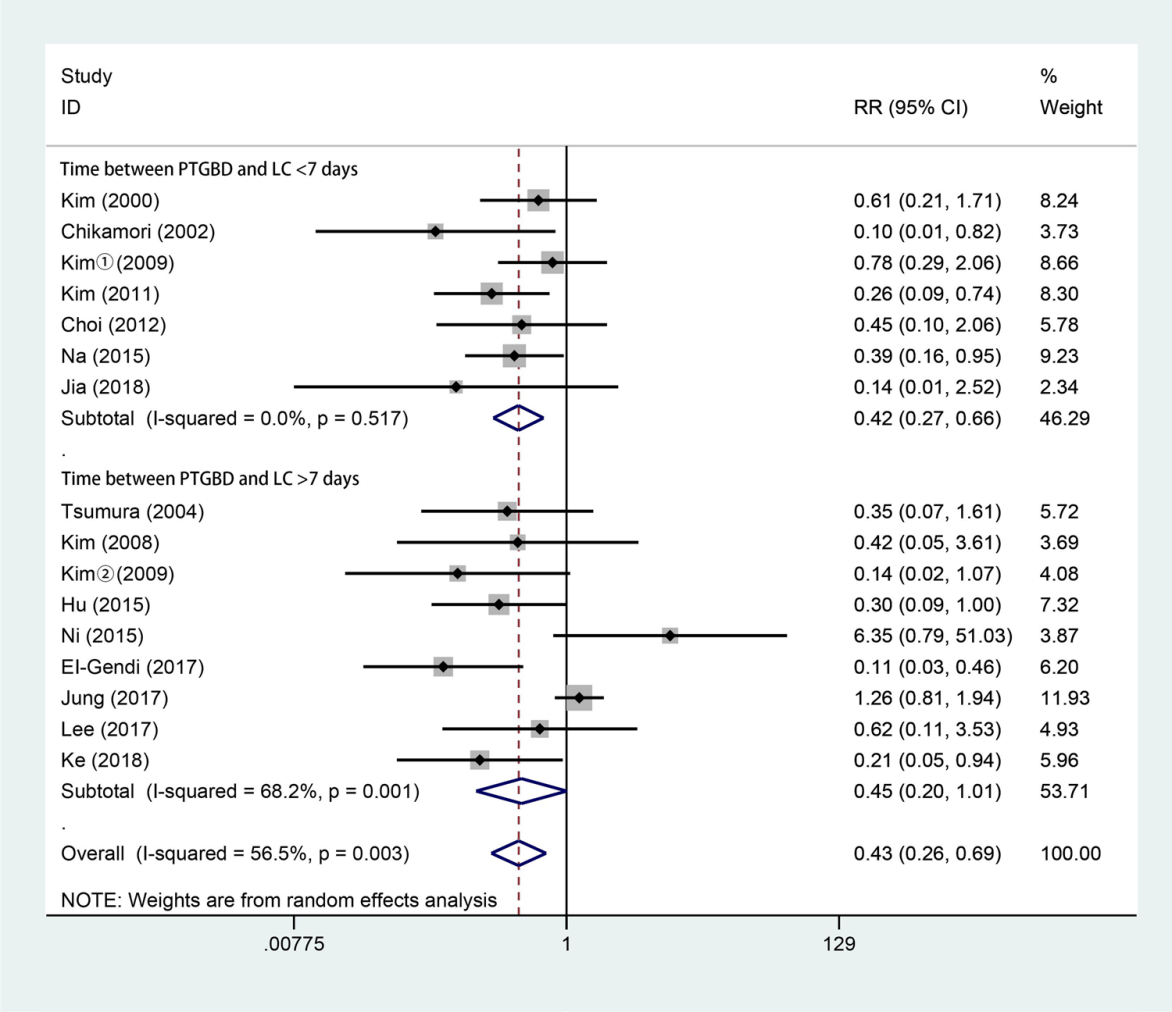
Forest plot of conversion rate of LC after PTGBD in patient with acute cholecystitis

All 15 studies [3, 4, 6, 10, 15–17, 28–35] reported the conversion rate from LC to OC. As shown in Fig. 3, 799 patients underwent LC after PTGBD, and 981 underwent ELC. According to the random-effects model, the pooled conversion rate of LC after PTGBD was favorable in the ELC group (RR 0.43; 95% CI 0.26–0.69; *P* = 0.001). There was a significant difference in heterogeneity among the studies (*Q* = 34.49; *P* for heterogeneity = 0.003; *I*^2^ = 56.5%).

### Total hospital stay

**Fig. 4:**
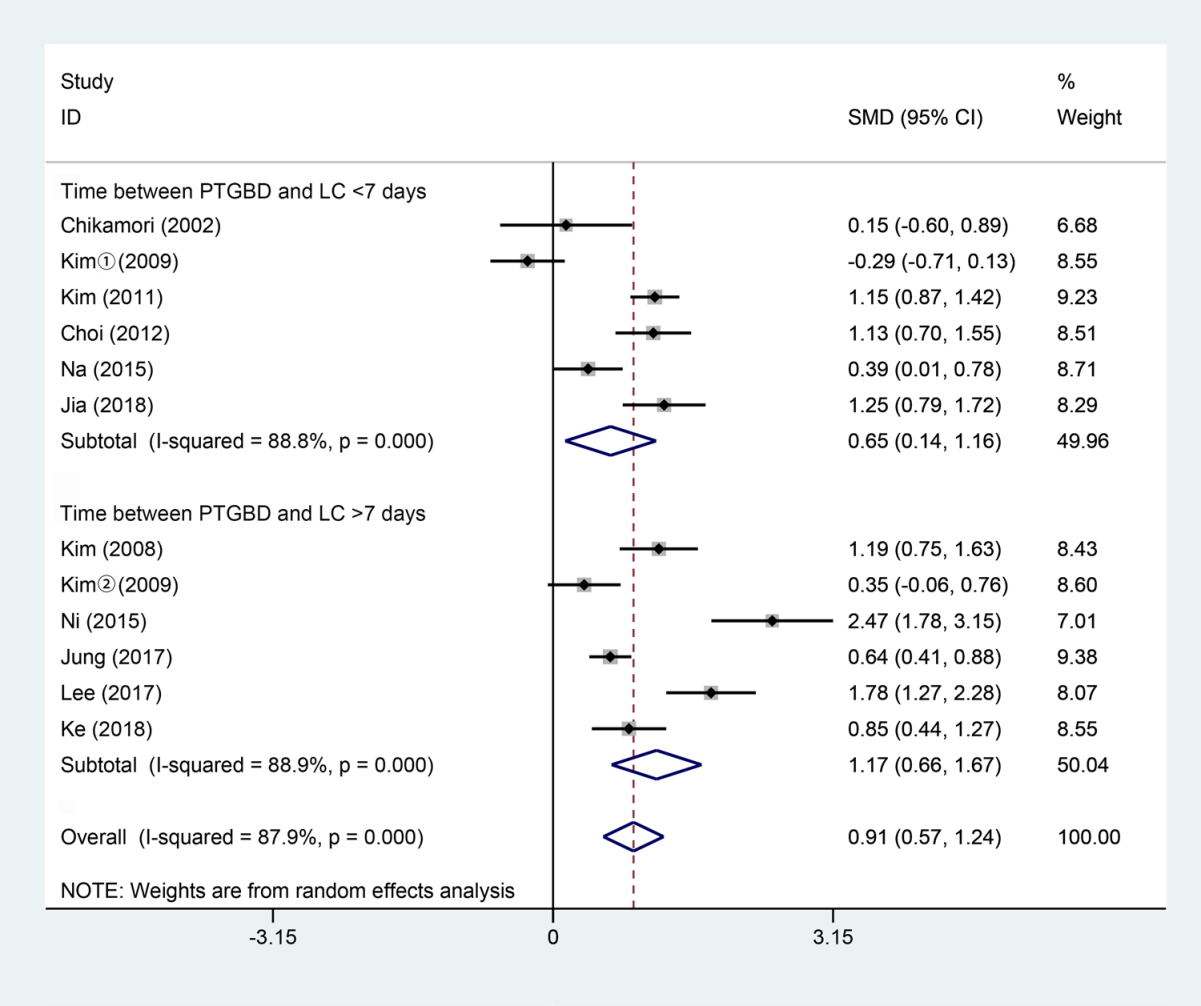
Forest plot about time of total hospital stay of LC after PTGBD in patient with acute cholecystitis

Eleven studies [3, 4, 6, 10, 15–17, 29–35] reported the total hospital stay. All of these studies were analyzed by the mean with standard deviation. As shown in Fig. 4, 602 patients underwent LC after PTGBD, and 813 underwent ELC. According to the random-effects model, the total hospital stay of LC after PTGBD group was longer than the ELC group (SMD 0.91; 95% CI 0.57–1.24; *P* < 0.001). There was a significant difference in heterogeneity among the studies (*Q* = 91.22; *P* for heterogeneity < 0.001; *I*^2^ = 87.9).

### Intraoperative blood loss

**Fig. 5:**
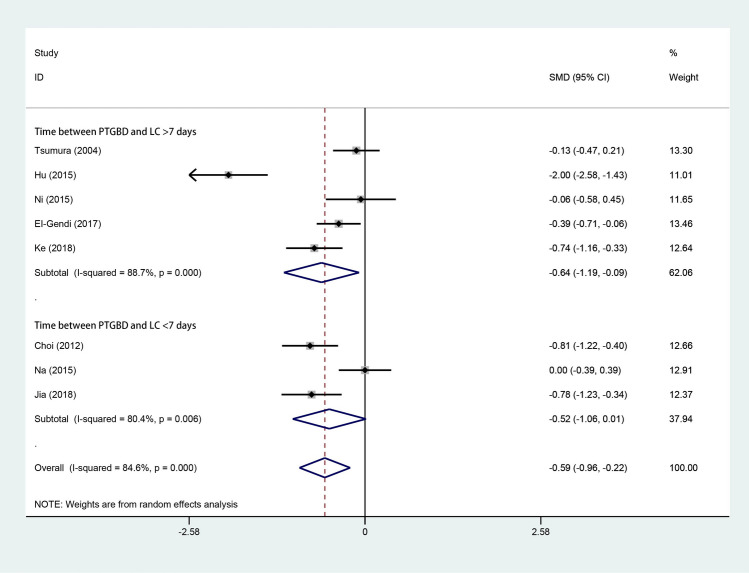
Forest plot of intraoperative blood loss of LC after PTGBD in patient with acute cholecystitis

Eight studies [3, 4, 10, 15, 17, 29–31] reported intraoperative blood loss. As shown in Fig. 5, 362 patients underwent LC after PTGBD, and 451 underwent ELC. According to the random-effects model, the intraoperative blood loss of LC in the PTGBD group was less than that in the ELC group (SMD − 0.59; 95% CI − 0.96 to − 0.22; *P* = 0.002). There was a significant difference in heterogeneity among the studies (*Q* = 45.50; *P* for heterogeneity < 0.001; *I*^2^ = 84.6%).

### Postoperative complications

**Fig. 6:**
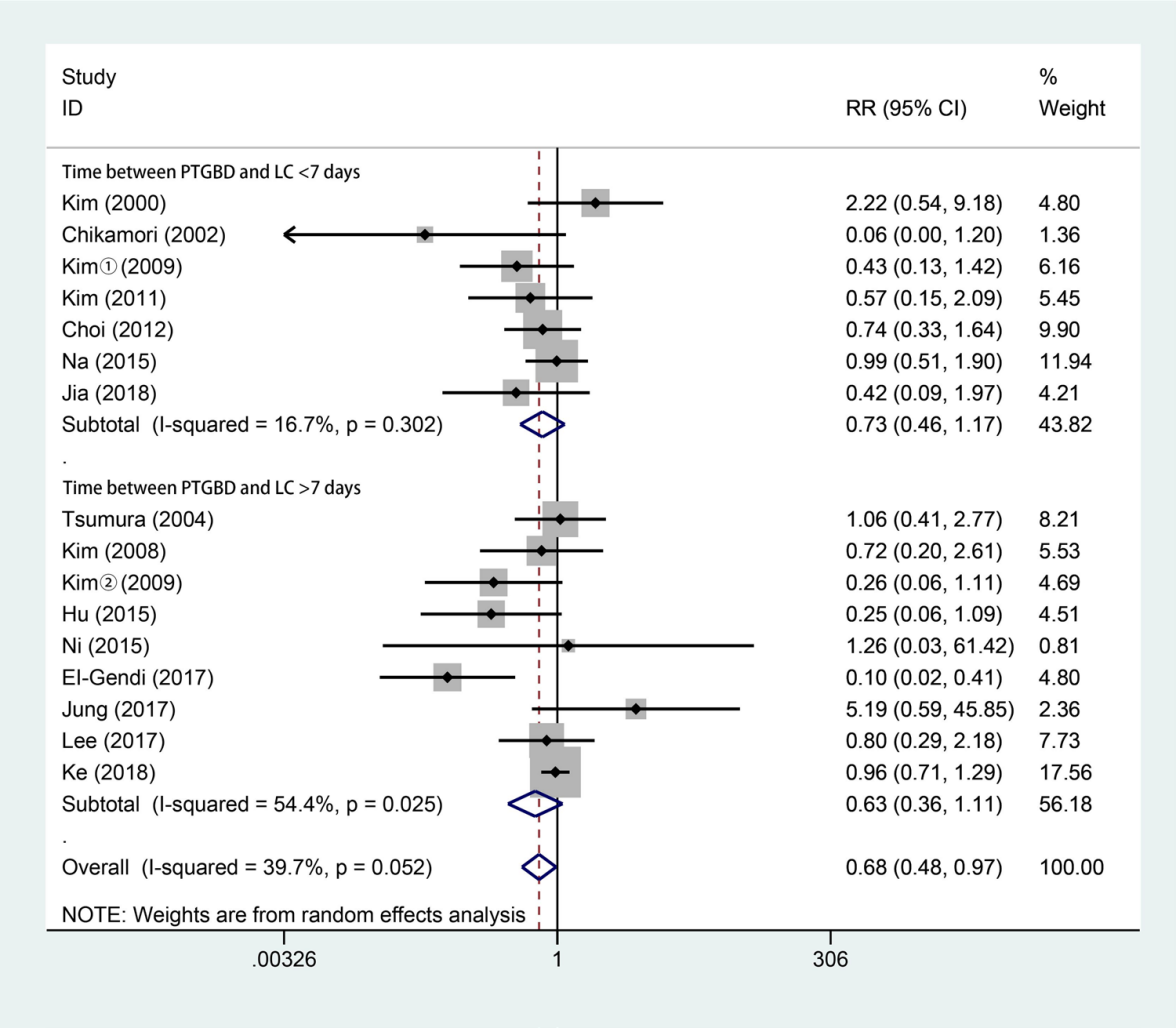
Forest plot of postoperative complications of LC after PTGBD in patient with acute cholecystitis

All studies [3, 4, 6, 10, 15–17, 28–35] reported postoperative complications, such as postoperative bleeding and persistent inflammation. As shown in Fig. 6, 799 patients underwent LC after PTGBD, and 981 underwent ELC. According to the random-effects model, the postoperative complications of LC in the PTGBD group were less severe than those in the ELC group (RR 0.68; 95% CI 0.48–0.97; *P* = 0.035). There was no significant difference in heterogeneity among the studies (*Q* = 24.88; *P* for heterogeneity = 0.052; *I*^2^ = 39.7%).

### Biliary leakage

**Fig. 7:**
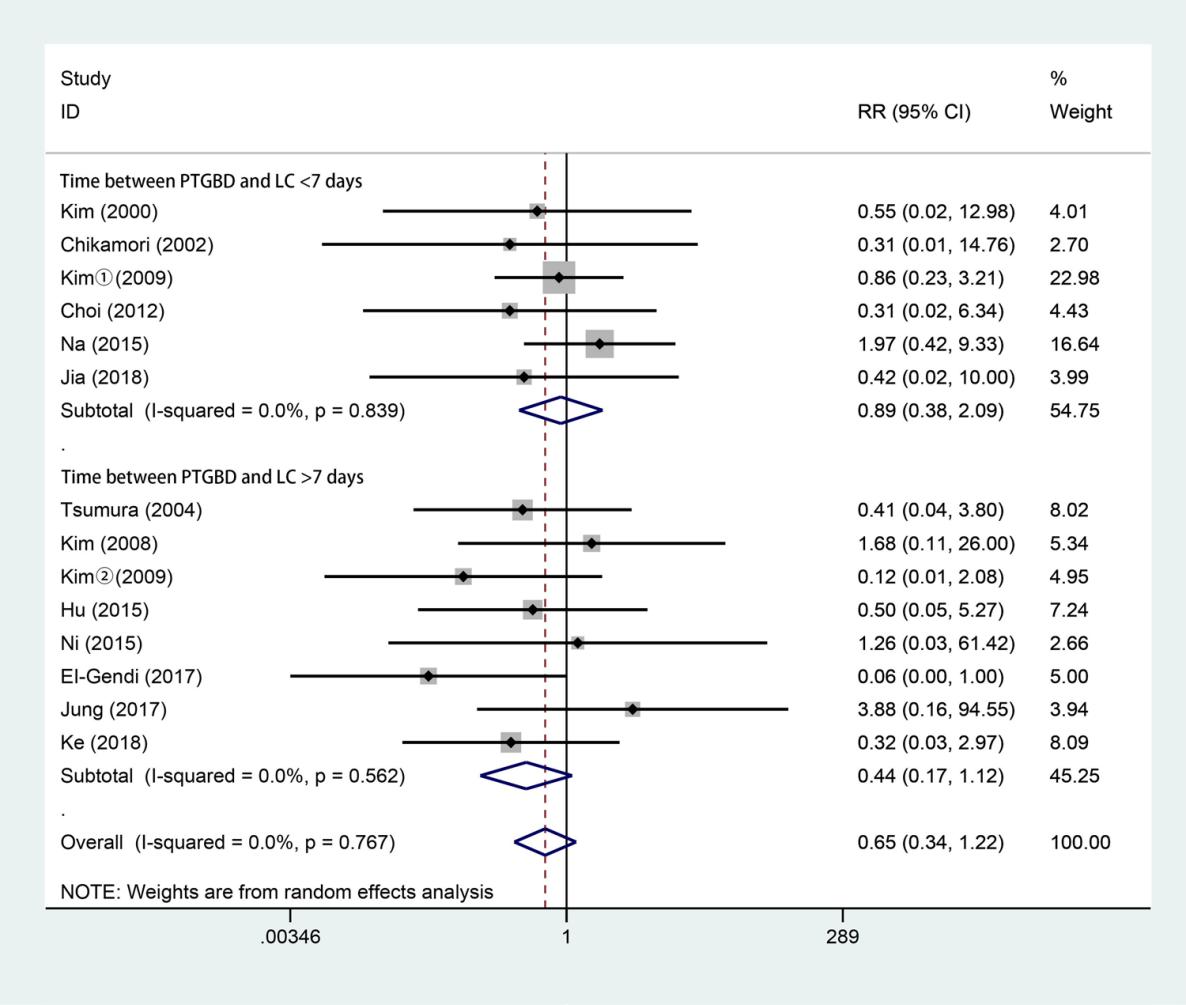
Forest plot of biliary leak of LC after PTGBD in patient with acute cholecystitis

Thirteen studies [3, 4, 6, 10, 15–17, 28–33] reported data on biliary leakage. As shown in Fig. 7, 658 patients underwent LC after PTGBD, and 793 underwent ELC. According to the random-effects model, there were no significant differences in biliary leakage between the two groups (RR 0.65; 95% CI 0.34 to 1.22; *P* = 0.175). There was no significant difference in heterogeneity among the studies (*Q* = 9.07; *P* for heterogeneity = 0.767; *I*^2^ = 0%).

### Mortality

**Fig. 8:**
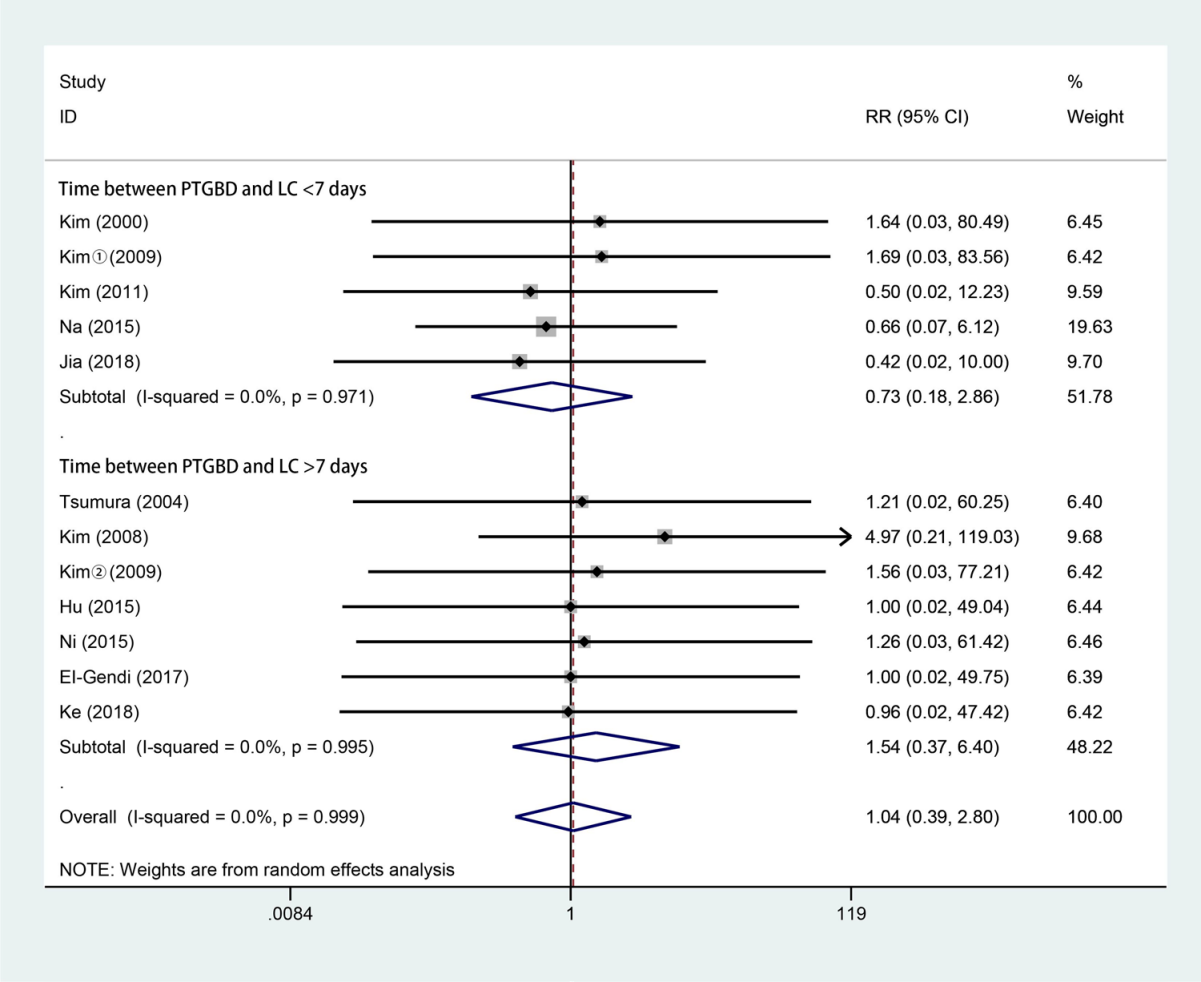
Forest plot of mortality of LC after PTGBD in patient with acute cholecystitis

Eleven studies [3, 4, 10, 17, 28–34] reported mortality rates. As shown in Fig. 8, 556 patients underwent LC after PTGBD, and 702 underwent ELC. According to the random-effects model, there were no significant differences in mortality between the two groups (RR 1.04; 95% CI 0.39–2.80; *P* = 0.933). There was no significant difference in heterogeneity among the studies (*Q* = 1.78; *P* for heterogeneity = 0.999; *I*^2^ = 0%).

### Publication bias analysis

**Fig. 9:**
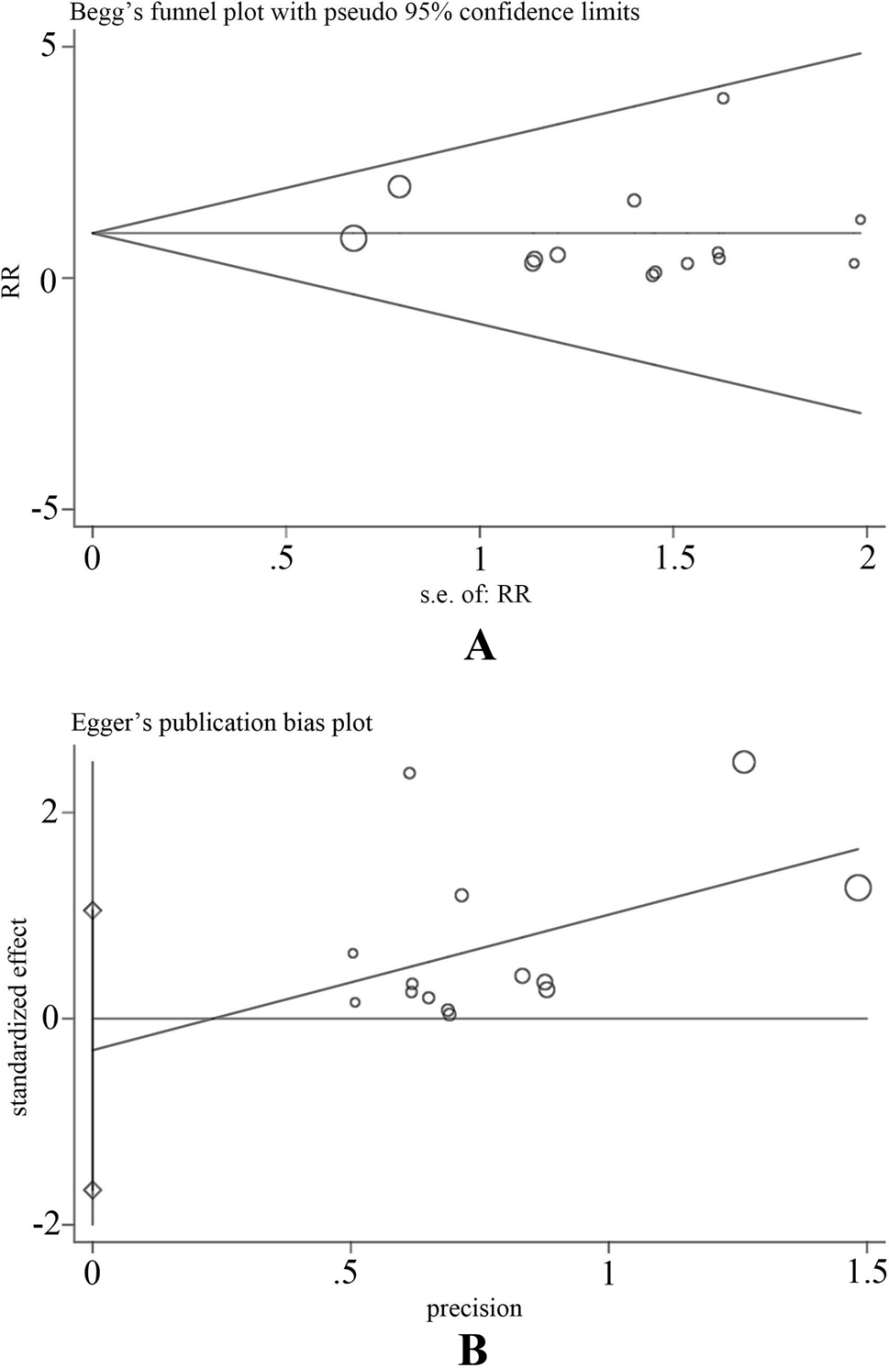
Begg’s funnel plot (**a**) and Egger’s test (**b**) of biliary leak were used to evaluate the publication bias

In this study, potential publication bias was investigated using Begg’s and Egger’s tests. The two plots for biliary leakage did not show obvious visual asymmetry (Fig. 9), and the *P* values of Egger’s test were also greater than 0.05 (*P* = 0.632). Moreover, the other *P* values of the index of the test were greater than 0.05 (data not shown). Therefore, there was no significant publication bias in this meta-analysis.

## Discussion

AC patients may present with a wide spectrum of disease severities linked to the operative difficulty, leading to the possibility of bile duct injury when AC patients undergo LC [2–4]. Several studies have also endorsed the usefulness of PTGBD for AC with a high success rate of efficacious drainage and a low complication rate, making it is a suitable treatment for AC patients [29, 30, 36]. However, no randomized controlled trials have been performed to clarify the advantages of both approaches. In addition, in clinical practice, many physicians decide their treatment strategies mostly based on local experience and personal preferences owing to the ambiguous indications for PTGBD [3, 34]. This leads to a controversial comparison between ELC and DLC after PTGBD. Given this background, we performed a meta-analysis to further clarify the safety and efficacy of ELC and PTGBD followed by DLC in AC patients.

The pooled analysis of the 15 included retrospective studies provided moderate quality evidence in favor of DLC after PTGBD for the treatment of acute cholecystitis. Our results showed that DLC after PTGBD had the advantages of a short operative time, a low conversion rate and limited intraoperative blood loss. However, there were no significant differences in several aspects including the postoperative length of hospital stay, postoperative complications, biliary leakage and mortality.

According to our analysis, DLC after PTGBD demonstrated a shorter operation duration than ELC (SMD − 0.51; 95% CI − 0.89 to − 0.13; *P* = 0.008). This finding could be related to the alleviation of local inflammation after PTGBD. As a minimally invasive procedure, PTGBD decompresses gallbladder distention and alleviates edema of the gallbladder wall and pericholecystic inflammation [6]. The clinical symptoms of AC in patients with good preoperative conditions can be immediately relieved by PTGBD [37]. According to a study from Hu et al. [30], 35 AC patients who underwent successful PTGBD had decompressed gallbladders and normal body temperatures within 72 h. Our result is also consistent with that of Chikamori et al. [16], who found that the duration of LC was shortened when cholecystectomy was performed as soon as possible after PTGBD. In addition, PTGBD can be employed for cholangiography to reveal the biliary tract anatomy and provide clear information about the surgical site, which might facilitate operative procedures [38].

Our analysis of the conversion rate suggested that DLC had a significantly lower potential to cause conversion to open surgery than ELC (RR 0.43; 95% CI 0.26–0.69; *P* = 0.001). Previous studies concluded that the root cause of conversion was repetitively progressive inflammation with distended and edematous walled gallbladders [39, 40]. The low conversion rate in the DLC group may be attributed to PTGBD, as PTGBD has the ability to relieve inflamed gallbladder adhesions [15]. On the other hand, emergency laparoscopic cholecystectomy is regarded as a challenging procedure in AC patients due to the frequent presence of adhesions around the acutely inflamed gallbladder and the high incidence of bile duct injuries [41]. In addition, in clinical practice, due to restrictions involving ethics, there might have been bias in terms of the selection of patients who had undergone PTGBD, as the patients in the DLC group had more severe preoperative inflammation, a higher risk of ASA classification and a poorer general condition than the patients in the ELC group [6, 15, 42]; DLC patients may have had severe inflammation and dense adhesions with an increased risk of conversion to laparotomy [43]. However, our conversion rate results still showed that DLC was superior to ELC, regardless of the effect of PTGBD. Our subgroup analysis also showed that there was no significant difference in the rate of conversion to open surgery when LC was performed within 7 days of or 7 days after PTGBD, similar to the results of previous studies [44, 45].

Our analysis of intraoperative hemorrhage revealed that DLC demonstrated less blood loss than ELC (SMD − 0.59; 95% CI − 0.96 to − 0.22; *P* = 0.002). PTGBD can immediately relieve the decompression of swollen gallbladders and pericholecystic inflammation to prevent the development of fibrosis in Calot’s triangle [37, 46]. With the help of PTGBD, the operation field of Calot’s triangle is much clearer, which facilitates laparoscopic cholecystectomy and reduces blood loss during the operation. In addition, surgeons have less information about patients who undergo ELC than what is routinely required. In contrast, during the preoperative period, the surgeon can identify the DLC patient’s underlying disease and the status of the biliary system, together with sufficient laboratory and radiological test results and surgical planning, which can enhance the patient’s safety [15, 34].

Our analysis of the hospital stay suggested that DLC after PTGBD had a significantly longer time of the total hospital stay than ELC (SMD 0.91; 95% CI 0.57 to 1.24; *P* < 0.001). Because of hospitalization for PTGBD, the time of total hospital stay was much longer in the PTGBD + DLC group. In clinical practice, patients in the PTGBD + DLC group may have poor general conditions and severe preoperative inflammation, which may need more time to retain the drainage [6, 15, 42]. Lo et al. [47] reported that PTGBD had the adverse outcome of a longer hospital stay for the management of gallbladder perforation, while urgent LC without PTGBD had similar surgical outcomes as that of elective LC with PTGBD. But previous studies suggested that AC patients treated with ELC may have a shorter postoperative hospital stay and lower hospital costs than those who underwent DLC without PTGBD [48–50].

Our analysis showed that there was no significant difference in terms of complications, biliary leakage or mortality. Apart from the complications caused by LC, PTGBD can also lead to complications related to the procedure. In the 49 patients with PTGBD from Ke’s study [17], there were 23 patients had postoperative complications after PTGBD and 12 cases among were associated with the drainage tube, such as bile leakage, hepatapostema and common bile duct stone. The complications caused by PTGBD may be associated with the long-time drainage and the underlying disease. However, patients in the EC group have higher incidence of respiratory failure and admission to the ICU, which contrarily indicates the effect of PTGBD on reducing severe complications after cholecystectomy in AC patients [17]. Besides, Giger et al. [43] analyzed more than 20,000 patients treated with LC and suggested that emergency surgery may be an independent risk factor for possible perioperative complications. In addition, Jackson et al. [51] and Jia et al. [29] suggested that postoperative complications may be associated with operative time and intraoperative blood loss, while our analysis showed a shorter operative time and less intraoperative blood loss in the DLC group than in the ELC group, which may imply a better outcome in terms of postoperative complications.

In our subgroup analysis, we divided studies into two groups based on the time interval between PTGBD and LC. Although it was not significant, probably because of the rough treatment of PTGBD time, the PTGBD time may affect the outcome of AC patients. Choi et al. [15] and Han et al. [52] reported that patients who underwent LC within 72 h after PTGBD had a worse outcome than those who underwent LC more than 72 h after PTGBD. These results can be explained by the fact that the short duration of PTGBD may cause the incompleteness of fistula formation around the PTGBD tube, and inflammation is not alleviated well enough for cholecystectomy [16].

Undoubtedly, there are several limitations in this meta-analysis. The quality of the included studies was deemed moderate, and all of the included studies were retrospective in nature, with the dearth of randomized controlled trials performed for ELC and PTGBD + DLC. Furthermore, limited by ethics, there was a bias in the selection of patients treated with PTGBD + DLC, as the patients in the DLC group may have worse conditions for cholecystectomy [6, 15, 42]. And patients who received PTGBD failed to undergo DLC are not reported in the literatures we included, which may lead to a publication bias in our meta-analysis. Finally, most of the included studies were performed in Asia, and the data regarding ELC and DLC in Europe and America were unclear. Randomized controlled trials and multicenter studies with a large sample size are needed to verify the outcomes of this meta-analysis.

In conclusion, our meta-analysis suggested that delayed cholecystectomy after PTGBD may be preferred over emergency cholecystectomy, as it had a shorter operative time, a lower conversion rate and less intraoperative blood loss. Therefore, delayed laparoscopic cholecystectomy after percutaneous transhepatic gallbladder drainage might be a good approach for patients with acute cholecystitis.
